# P-1646. Return to Work or School among Adults After Symptomatic SARS-CoV-2 Infection

**DOI:** 10.1093/ofid/ofaf695.1821

**Published:** 2026-01-11

**Authors:** Ning Zhang, Katie R Mollan, Dinelka Nanayakkara, Jessica Keys, Becky Straub, David A Wohl, William A Fischer

**Affiliations:** UNC Chapel Hill, Chapel Hill, NC; UNC Gillings School of Public Health, Chapel HIll, North Carolina; UNC Chapel Hill, Chapel Hill, NC; UNC Chapel Hill, Chapel Hill, NC; UNC Chapel Hill, Chapel Hill, NC; University of North Carolina at Chapel Hill School of Medicine, Chapel Hill, North Carolina; Institute for Global Health and Infectious Diseases, the University of North Carolina, Chapel Hill, North Carolina

## Abstract

**Background:**

Return to work or school following acute COVID-19 infection is a key recovery milestone. Understanding when adults resume their occupational activities has important implications for the US economy and public health response.

**Figure 1.**
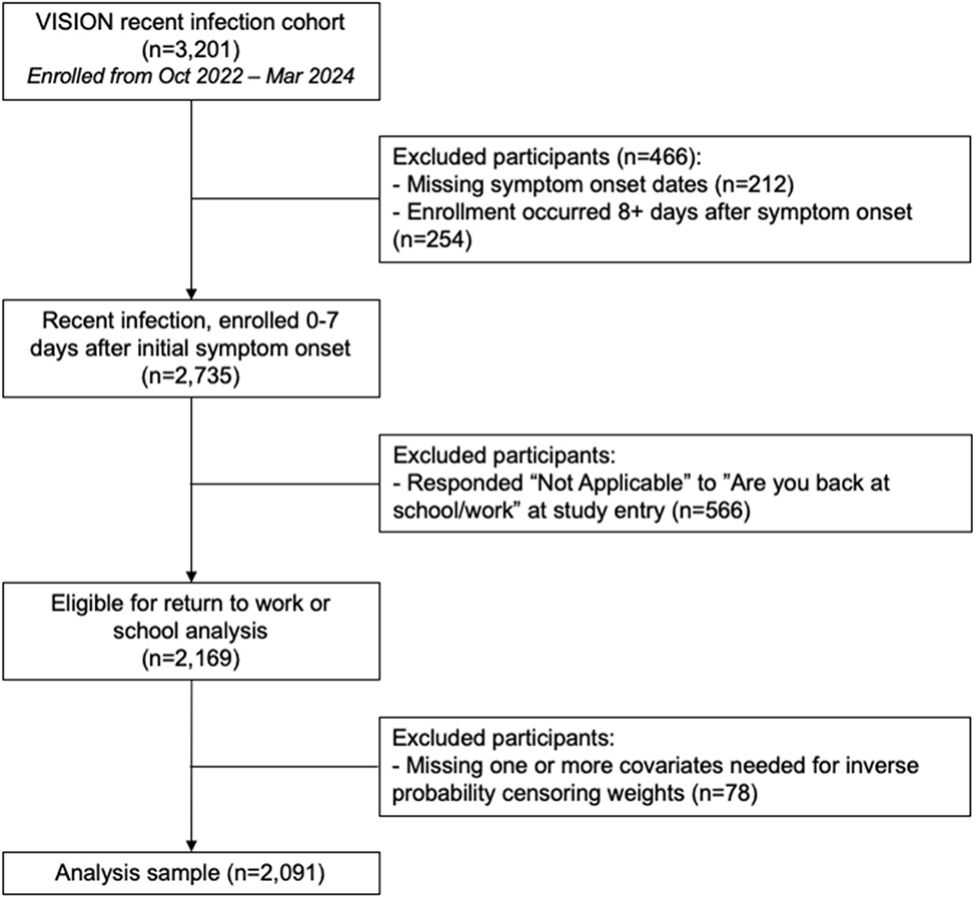
CONSORT diagram of participant inclusion in the VISION recent infection cohort for return to work or school.

**Figure 2.**
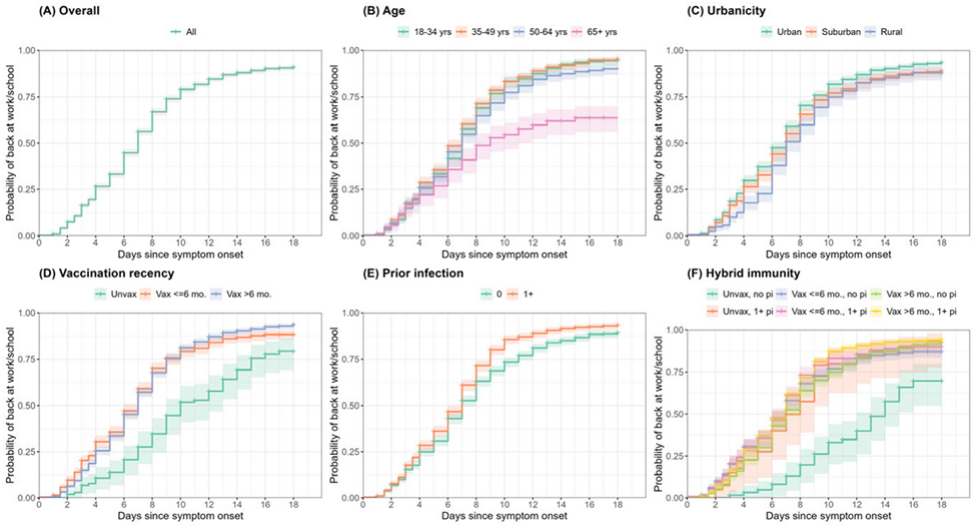
Weighted Kaplan-Meier curves of returning to work or school in the overall sample and stratified by age, residential urbanicity, vaccination recency, prior infection, and hybrid immunity. Hybrid immunity refers to the combination of vaccination recency and prior infection. 95% CIs were estimated using robust standard errors.

**Methods:**

We examined patterns of returning to work/school among adults with symptomatic COVID-19 who tested SARS-CoV-2 positive within 0-7 days of study enrollment participating in the VISION study, a large prospective cohort of North Carolina residents with SARS-CoV-2. Analyses were restricted to participants who enrolled within 0-7 days after symptom onset and reported at study entry that return to work/school was applicable to them. Participants were asked daily via an online questionnaire (study days 1-14) if they were back at work/school. We estimated the probability of returning to work/school using inverse probability censoring weighted Kaplan-Meier curves. To summarize the timing of return to work/school, we compared 10-day restricted mean survival time (average time not returned) by baseline characteristics, including bivariate analyses of: age, sex, race, ethnicity, body mass index, vaccination recency, prior infection, hybrid immunity, and residential urbanicity.

**Table 1.**
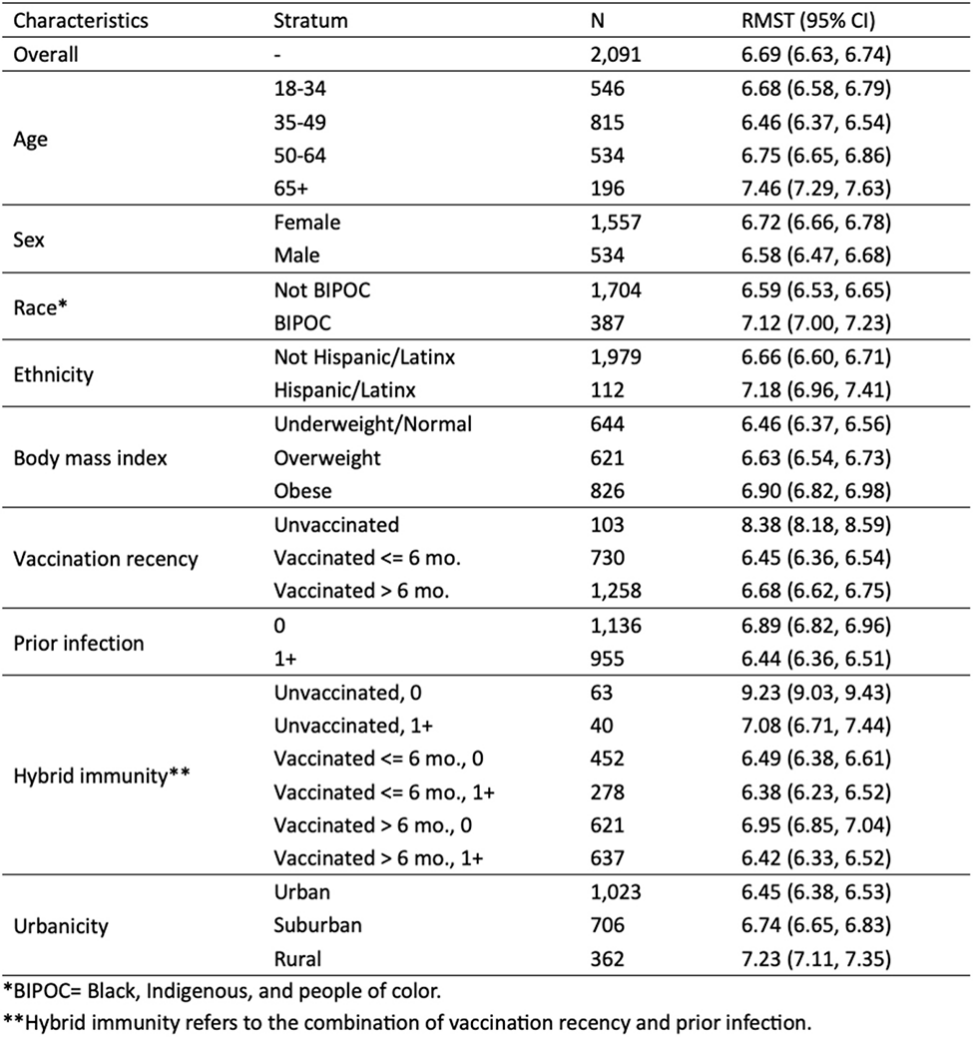
Restricted mean survival time (RMST) for days not at work or school during the first 10 days after symptom onset.

**Results:**

Among 2,169 eligible participants enrolled October 2022 to March 2024 (Figure 1), the median age was 43 years (IQR: 34, 54); 35% received their last SARS-CoV-2 vaccine ≤6 months ago, and 54% were experiencing their first known SARS-CoV-2 infection. The estimated probabilities of returning to work/school by day 7 and day 10 after symptom onset were 56% (95% CI 54-58%) and 79% (95% CI 77-81%), respectively (Figure 2A). During the first 10 days after symptom onset, unvaccinated adults with no prior infections had the slowest return, with an average of 9.2 (95% CI 9.0-9.4) days out of work/school (Table 1), whereas those with at least one prior infection or vaccination took an average of 6.4 to 7.1 days to return to work/school. The fastest return was observed among those vaccinated and with prior infection(s).

**Conclusion:**

Return to work or school occurred within two weeks of symptom onset for most adults in NC. However, unvaccinated people and adults without prior infection took longer to return to work/school.

**Disclosures:**

Katie R. Mollan, MS, Gilead Sciences: Grant/Research Support David A. Wohl, M.D., EMD Serono: Advisor/Consultant|EMD Serono: Honoraria|Gilead Sciences: Advisor/Consultant|Gilead Sciences: Honoraria|Merck: Advisor/Consultant|Merck: Honoraria|Regeneron: Advisor/Consultant|Regeneron: Honoraria|Theratechnologies: Advisor/Consultant|Theratechnologies: Honoraria|ViiV: Advisor/Consultant|ViiV: Honoraria William A. Fischer, II, MD, Inhalon: Advisor/Consultant

